# Internet-Based Cognitive Behavioral Therapy With Real-Time Therapist Support via Videoconference for Patients With Obsessive-Compulsive Disorder, Panic Disorder, and Social Anxiety Disorder: Pilot Single-Arm Trial

**DOI:** 10.2196/12091

**Published:** 2018-12-17

**Authors:** Kazuki Matsumoto, Chihiro Sutoh, Kenichi Asano, Yoichi Seki, Yuko Urao, Mizue Yokoo, Rieko Takanashi, Tokiko Yoshida, Mari Tanaka, Remi Noguchi, Shinobu Nagata, Keiko Oshiro, Noriko Numata, Motohisa Hirose, Kensuke Yoshimura, Kazue Nagai, Yasunori Sato, Taishiro Kishimoto, Akiko Nakagawa, Eiji Shimizu

**Affiliations:** 1 United Graduate School of Child Development Osaka University, Kanazawa University, Hamamatsu University School of Medicine, Chiba University and University of Fukui Osaka Japan; 2 Research Center for Child Mental Development Graduate School of Medicine Chiba University Chiba Japan; 3 Department of Cognitive Behavioral Physiology Graduate School of Medicine Chiba University Chiba Japan; 4 Research Center for Medical Economics Administration Chiba University Hospital Chiba Japan; 5 Reseach and Education Center of Health Sciences Gunma University Graduate School of Health Sciences Gunma Japan; 6 Department of Preventive Medicine and Public Health Keio University Tokyo Japan; 7 Department of Neuropsychiatry Keio University School of Medicine Tokyo Japan

**Keywords:** clinical trial, cognitive behavioral therapy, feasibility study, obsessive-compulsive disorder, panic disorder, social anxiety disorder, videoconference

## Abstract

**Background:**

Cognitive behavioral therapy (CBT) is the first-line treatment for adults with obsessive-compulsive disorder (OCD), panic disorder (PD), and social anxiety disorder (SAD). Patients in rural areas can access CBT via the internet. The effectiveness of internet-delivered cognitive behavioral therapy (ICBT) has been consistently shown, but no clinical studies have demonstrated the feasibility of ICBT with real-time therapist support via videoconference for OCD, PD, and SAD at the same time.

**Objectives:**

This study aimed to evaluate the feasibility of videoconference-delivered CBT for patients with OCD, PD, or SAD.

**Methods:**

A total of 30 Japanese participants (mean age 35.4 years, SD 9.2) with OCD, SAD, or PD received 16 sessions of individualized videoconference-delivered CBT with real-time support of a therapist, using tablet personal computer (Apple iPad Mini 2). Treatment involved individualized CBT formulations specific to the presenting diagnosis; all sessions were provided by the same therapist. The primary outcomes were reduction in symptomatology, using the Yale-Brown obsessive-compulsive scale (Y-BOCS) for OCD, Panic Disorder Severity Scale (PDSS) for PD, and Liebowitz Social Anxiety Scale (LSAS) for SAD. The secondary outcomes included the EuroQol-5 Dimension (EQ-5D) for Quality of Life, the Patient Health Questionnaire (PHQ-9) for depression, the Generalized Anxiety Disorder (GAD-7) questionnaire for anxiety, and Working Alliance Inventory-Short Form (WAI-SF). All primary outcomes were assessed at baseline and at weeks 1 (baseline), 8 (midintervention), and 16 (postintervention) face-to-face during therapy. The occurrence of adverse events was observed after each session. For the primary analysis comparing between pre- and posttreatments, the participants’ points and 95% CIs were estimated by the paired *t* tests with the change between pre- and posttreatment.

**Results:**

A significant reduction in symptom of obsession-compulsion (Y-BOCS=−6.2; Cohen *d*=0.74; 95% CI −9.4 to −3.0, *P*=.002), panic (PDSS=−5.6; Cohen *d*=0.89; 95% CI −9.83 to −1.37; *P*=.02), social anxiety (LSAS=−33.6; Cohen *d*=1.10; 95% CI −59.62 to −7.49, *P*=.02) were observed. In addition, depression (PHQ-9=−1.72; Cohen *d*=0.27; 95% CI −3.26 to −0.19; *P*=.03) and general anxiety (GAD-7=−3.03; Cohen *d*=0.61; 95% CI −4.57 to −1.49, *P*<.001) were significantly improved. Although there were no significant changes at 16 weeks from baseline in EQ-5D (0.0336; Cohen *d*=-0.202; 95% CI −0.0198 to 0.00869; *P*=.21), there were high therapeutic alliance (ie, WAI-SF) scores (from 68.0 to 73.7) throughout treatment, which significantly increased (4.14; 95% CI 1.24 to 7.04; *P*=.007). Of the participants, 86% (25/29) were satisfied with videoconference-delivered CBT, and 83% (24/29) preferred videoconference-delivered CBT to face-to-face CBT. An adverse event occurred to a patient with SAD; the incidence was 3% (1/30).

**Conclusions:**

Videoconference-delivered CBT for patients with OCD, SAD, and SAD may be feasible and acceptable.

## Introduction

### Background

Obsessive-compulsive disorder (OCD), panic disorder (PD), and social anxiety disorder (SAD) are the most common mental disorders and incur a huge burden throughout the lifespan [1-4]. Cognitive behavioral therapy (CBT) has been found to be effective in treating all of the 3 disorders [5-16]. Although CBT is an effective treatment, it is difficult for all patients to receive CBT because of problems of access to treatment such as expensive specialized medical treatment, lack of therapists, and uneven urban distribution. Known as telemental health, a new therapeutic approach, born out of technological innovation after the internet revolution, has solved these problems. A patient can now receive treatment from remote distance via the internet, regardless of physical or psychological barriers such as time, distance, and stigma from remote distance by the internet [17,18]. For example, in internet-delivered CBT (ICBT), users can receive their programmed treatment at any time, 24 hours a day. In addition, the therapist’s involvement with the patient can be reduced, optimizing treatment costs. Furthermore, a therapist guide can be employed via remote treatment, by using videoconference in real time, without compromising linguistic and nonverbal communication with the patient as much as possible.

ICBT has been shown to be effective for OCD, PD, and SAD from several randomized controlled trials [19-35]. A recent systematic review and meta-analyses using 20 studies comparing ICBTs and face-to-face CBTs showed that ICBT and face-to-face treatment produced equivalent overall effects [36]. Target diseases in this meta-analysis included SAD, PD, and depression. In the other meta-analyses, comparing the effectiveness of ICBT and face-to-face CBT with anxiety disorders including OCD (defined according to the Diagnostic and Statistical Manual of Mental Disorder III [37], III-R [38], IV [39], and IV-TR or the International Classification of Diseases 9 or 10 [40-42]), there were no clear differences between them [43]. ICBT can be roughly divided into the 3 categories depending on how the therapist participates in the program [44]: programs with no therapist assistance [45]; programs with assistance, where the therapists assistance is minimal [46]; and live conversations on the internet, where the therapist is fully involved using videoconference [47,48]. Although the effectiveness of ICBT has been suggested, there is little knowledge about ICBT including videoconference. In telemental health, which is an important theme when considering the optimization of social resources, the effectiveness of ICBT should be examined based on the therapist’s degree of involvement. In this study, we examined the feasibility of ICBT with real-time videoconference, where the therapists were fully involved in treatment.

### Videoconference-Delivered Cognitive Behavioral Therapy

In videoconference-delivered CBT, the therapist and patient use video and audio links to have a therapeutic conversation, as in face-to-face CBT, which includes nonverbal information such as expressions, body language, voice volume, and tone. Therefore, among the ICBT options, this allows the patient and the therapist to have the strongest therapeutic relevance. In a situation where real-time communication with the patient is important, it is considered to be a powerful approach; for example, when the therapist can enhance motivation through role playing by performing internal sensory exposure in front of a patient with serious panic PD.

Mental health services for remote populations via videoconferences have yielded high satisfaction [17,49-52]. A systematic review of videoconference-delivered CBT trials, using 20 controlled studies, uncontrolled studies, case series, and case studies for anxiety disorders including posttraumatic stress disorder (PTSD) using the criteria of the Diagnostic and Statistical Manual of Mental Disorders (DSM-IV-TR), suggested a medium to large effect size [53]. At the same time, there were no clinical studies about videoconference-delivered CBT for all of OCD, PD, and SAD. Comorbidity has often been seen between OCD, PD, and SAD [54]; clinical trials of CBT with additional remote systems to determine videoconference-delivered CBT adaptation to normal clinical scenes are thus meaningful. A randomized controlled trial compared OCD using professional videoconferencing equipment (n=10) with self-help (n=10) and wait-list (n=10) controls [51]. The results indicated large effect sizes (Cohen *d*=2.1-2.5); in addition, 60% (6/10) of participants in the videoconference condition achieved clinically significant changes at posttreatment and 50% (5/10) did so at a 3-month follow-up. Another controlled trial compared manualized CBT for PD and agoraphobia by videoconference (n=11) with in-person CBT (n=10) [52]. The results indicated no significant difference between the conditions, but 81% (9/11) of participants in the videoconferencing condition were panic-free at posttreatment and 91% (10/11) at a 9-month follow-up; this indicated a very large effect size for all panic and agoraphobia symptoms. An uncontrolled trial, using 24 participants with SAD, applied acceptance-based behavioral therapy via videoconference [50] and found that 54% (13/24) of participants did not meet DSM-IV-TR criteria for SAD at posttreatment. Large effect sizes for all social anxiety symptoms (Cohen *d*=1.23-1.91) were obtained at post-therapy and at a 3-month follow-up. In addition, it is known that videoconference-delivered therapy can develop a similarly strong therapeutic alliance with psychotherapy clients as in-person therapy [49,55].

Nevertheless, during this trial, the only national university hospital with a CBT specialized outpatient in Japan was at our facility alone, the Chiba University; most patients do not have access to this treatment. For patients in Japan to receive CBT via the internet, it was necessary to examine the feasibility of videoconference-delivered CBT.

### Objective of This Study

Previous studies have consistently demonstrated the effectiveness of videoconference-delivered CBT, but they have small samples sizes and were conducted in Western countries. Therefore, this pilot study utilized an open trial to examine the feasibility and preliminary effectiveness of in-home CBT via videoconference for Japanese adults with OCD [49], PD, and SAD. It was hypothesized that videoconference-delivered CBT would be effective to reduce the symptomatology for each disorder from pre- to posttreatment and acceptable to Japanese participants.

## Methods

### Study Design

This study was conducted as a single-arm, open trial at the academic outpatient clinic of the Cognitive Behavioral Therapy Center of Chiba University Hospital between March 2017 and March 2018. Since this trial was the first to employ an individual videoconference-delivered CBT intervention design against OCD, PD and SAD in Japan, a single-arm trial examining baseline predictors rather than effectiveness was considered to be an appropriate design [56].

### Ethics and Dissemination

This trial was approved by the Institutional Review Board of Chiba University Hospital (reference number: G28038). The clinical trial registration number was UMIN000026609.

If an individual wished to participate in the trial, they had to contact the study trial office, where they were informed about the study objectives and asked to confirm whether they were willing to participate. Furthermore, they were assured absolute anonymity. They had to fill out an informed consent form for participation in this study. All participants were informed that they could continue receiving conventional drug treatments from their primary doctors. We practiced videoconferencing twice with participants.

### Participants and Eligibility Criteria

The study participants were recruited through posters and leaflets placed at medical institutions in Chiba Prefecture, through the official Web-based advertisements at the Cognitive Behavioral Therapy Center of Chiba University Hospital and by referrals from their primary care doctors or psychiatrists. After email or telephone screening through Web-based app, the participants visited our center and were diagnosed with OCD, SAD, or PD using the Mini-International Neuropsychiatric Interview [57,58].

Inclusion criteria for this study included informed consent to participate in the study; having a primary diagnosis of OCD, PD, or SAD; aged between 19 and 65 years, and having access to the internet at home. Comorbid mental disorders—including major depressive disorder, other anxiety disorders, and eating disorders—were permitted if they were clearly secondary, considering that this trial should reflect routine clinical practice. The exclusion criteria were organic brain damage, dementia, psychotic disorders, serious drug dependence, recurrent suicidal and antisocial behaviors, and severe somatic conditions. Participants, who used psychotropic drugs, including selective serotonin reuptake inhibitors and benzodiazepines, were asked to report all of changes regarding pharmacotherapy during the study period.

### Intervention

The participants entered a Web conference room by clicking a URL in an email sent from their therapists. The intervention was conducted at a 50-min session once a week for 16 weeks. The modules were derived from previous studies on in-person CBT for OCD [59], PD [60], and SAD [61] in Japan and included psychoeducation, exposure exercises, behavioral experiments, and homework assignments.

### Therapists and Therapy Quality Control

Videoconference-delivered CBT was delivered by 12 therapists, who were experienced in face-to-face CBT for patients with OCD, PD, and/or SAD (including 7 clinical psychologists, 2 psychiatric social workers, 1 nurse, 1 psychiatric pharmacist, and 1 psychiatrist). Therapists were trained in CBT programs for patients with OCD, PD, and SAD and attended weekly group-supervision sessions with other therapists as well as undergoing individual supervision by a senior supervisor. All therapists had completed a CBT training course (Chiba Improving Access to Psychological Therapies project: Chiba-IAPT) [62]. Of the therapists, 6 were female, with an average age of 43.5 years (SD 7.5) and an average of 2.2 years of clinical experience (SD 6.4) at the beginning of the study. Senior supervisors assessed the quality of the videoconference-delivered CBT sessions using the Cognitive Therapy Scale-Revised [63,64], a revision of the Cognitive Therapy Scale designed by Young and Beck (unpublished data [65,66]).

### Visual Aids

The use of visual aids facilitates the learning process by enhancing motivation and understanding of complex matters [67]. Therefore, visual aids were used in each program to enhance the participants’ understanding. The visual aids consisted of several slides including key concepts of CBT. The therapists conducted CBT sessions with the visual aids by using the screen-sharing function of the videoconference software, sending them to the participants as password-protected homework slides by email after each session.

### Hardware

The therapists used 2 Surface Pro 2 computers―2-in-1 detachable produced by Microsoft , running Windows 10 Pro (Microsoft Corporation, US). The display size was 10.6 inches and a resolution of 1920×1080 pixels. Each participant was lent an iPad Mini 2 (Apple Inc, US) with a 7.9-inch display and a 2048×1536-pixel resolution.

### Software for Videoconference

A total of 3 licenses for videoconference software (Cisco WebEx, Milpitas, CA, USA) were used in this trial. This system has been awarded ISO27001 certification (regarding handling of information security) and SSAE16 (Statement of Standards for Attestation Engagements No. 16: former SAS 70) compliance certification (issued by a third party). It also complies with the United States *Health Insurance Portability and Accountability Act*. WebEx’s use of a switching network along with a 128-bit Secure Sockets Layer encryption and public key infrastructure is regarded by the Japan Ministry of Health, Labor and Welfare to have solved the problem of safety, as reported in their “Guidelines on Safety Management of Medical Information System Version 4.3” in March 2016 [68]. Since the stability and safety of the software are excellent and it sufficiently protects personal information, we judged WebEx to be sufficiently reliable for this study.

### Measures of Primary Outcomes

The most commonly used scales to measure symptoms of each disorder were used as follows: the Yale-Brown Obsessive-Compulsive Scale (Y-BOCS) for OCD symptoms [69,70], the Panic Disorder Severity Scale (PDSS) for PD symptoms [71,72], and the Liebowitz Social Anxiety Scale (LSAS) for SAD symptoms [73,74].

To calculate responsiveness to treatment and the remission rate after the intervention, the criteria of the previous studies regarding the severity rating scale of each disorder was used [62,64,65]. For OCD, treatment response was defined as a 35% or greater reduction in the total Y-BOCS score, and remission was defined as a final Y-BOCS score of ≤14 [75]. For PD, treatment response was defined as a 40% or greater reduction in total PDSS score, and remission was defined as a final PDSS score of ≤5 [76]. For SAD, treatment response was defined as a 31% or greater reduction in total LSAS score, and remission was defined as a final LSAS score of ≤36 [73].

### Measures of Secondary Outcomes

We used the EuroQol-5 Dimension (EQ-5D) to measure health-related quality of life [77,78]. This trial measured the psychological bond between therapist and participant using the Working Alliance Inventory-Short Form (WAI-SF) [79], depressive symptoms using the Patient Health Questionnaire-9 (PHQ-9) [80,81], and generalized anxiety symptoms using the Generalized Anxiety Disorder-7 (GAD-7) [82,83]. The definition of the response in PHQ-9 and GAD-7 was defined as a 50% reduction in total score. We used 7-point Likert scale format to measure participants’ satisfaction about videoconference-delivered CBT as follows: “Very dissatisfied,” “Dissatisfied,” “Slightly dissatisfied,” “Neutral,” “Slightly satisfied,” “Satisfied,” and “Very satisfied.” In addition, the participants were asked about preference of videoconference-delivered CBT or face-to-face CBT as follows: “If you could choose in the future, would you wish to receive treatment with either face-to-face or videoconference CBT?” Participants answered using a 7-point Likert scale as follows: “Clearly prefer face-to-face,” “Prefer face-to-face,” “Slightly prefer face-to-face,” “Neutral,” “Slightly prefer videoconference-delivered CBT,” “Prefer videoconference-delivered CBT,” and “Clearly prefer videoconference-delivered CBT.”

### Data Setting and Locations

Participant and therapists used the Numbers app for iOS to run the digital questionnaires, and the therapist asked each participant to answer them by themselves on the tablet PC at weeks 1, 8, and 16. Each participant sent an email with the completed questionnaires of all primary outcomes (Y-BOCS, PDSS, and LSAS) and part of secondary outcomes (EQ-5D, PHQ-9, and GAD-7) to their therapist before each session and sent an email with the completed questionnaires of the secondary outcomes (WAI-SF and satisfaction/preference) attached after session. The therapist checked outcomes and evaluated the symptoms during the session, collaborating with the participant. The collected data were registered to the server of DATATRAK ONE (DATATRAK International Inc, US) as Web case registration system by the lead author and managed by the data management office of Chiba University. This study adhered to the CONSORT-EHEALTH guidelines for improving and standardizing the report of Web-based and mobile health interventions [84].

### Adverse Events

To confirm the occurrence of adverse events after intervention, the therapist asked the patient about their physical and mental condition at the end of each session and instructed all participants to report all adverse events by email.

### Statistical Analysis

Statistical analysis and reporting of this trial were conducted in accordance with the CONSORT-EHEALTH guidelines [84]. All statistical analyses were described in the statistical analysis plan, which was fixed before the database lock. All efficacy analyses were primarily based on the full analysis set, which included all patients who had received at least one session of the videoconference-delivered CBT treatment. For baseline variables, summary statistics were constructed, employing frequencies and proportions for categorical data and means and SDs for continuous variables. Baseline variables were compared using the Fisher exact test for categorical outcomes and the unpaired *t* test for continuous variables. For the primary analysis comparing between pre- and posttreatments, the points and their 95% CIs were estimated by the paired *t* tests with the change at week 16 from baseline in EQ-5D index scores for all of the patients in Y-BOCS for OCD, PDSS for PD, and in LSAS for SAD. For comparison among the 3 disorders, a one-way analysis of variance was used. Analyses of secondary outcomes were performed in the same manner as the primary analysis.

In addition, we calculated Cohen *d* pre-post effect sizes by calculating the mean differences between pre- and posttreatments, dividing by the pooled SDs. We also adopted the criteria that a Cohen *d* of >0.20 was a small effect, that of >0.50 was a medium effect, and that of >0.80 was a large effect [85]. All *P* values were two-sided; a value of *P*<.05 was considered statistically significant. Statistical analyses were performed using SAS software version 9.4 (SAS Institute, Cary, North Carolina, USA) and the R statistical program version 2.13 (The R Foundation, Vienna, Austria).

## Results

### Recruitment

**Figure 1:**
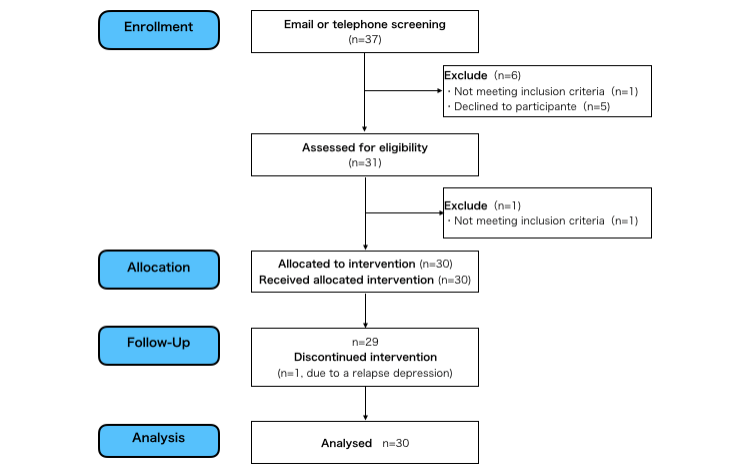
Participant flow.

Figure 1 shows the participant flow. A total of 37 patients applied to participate through our website. After email or telephone screening, 6 patients were excluded; 1 did not meet one of the inclusion criteria due to epilepsy and 5 declined to participate because of long distance to our hospital. After the screening, 31 attended the face-to-face baseline assessment, and one did not meet one of the inclusion criteria due to high risk of suicide. Finally, 30 patients were enrolled to the study.

### Attrition

Of the participants eligible to take part in the study, 1 SAD participant with major depressive disorder dropped out after 9 sessions because of worsening of his depressive state. The remaining 97% (29/30) completed the full course of videoconference-delivered CBT. All data at point each assessment (screening, session 1, session 8, and session 16) were statistically analyzed.

### Clinical and Demographic Characteristics

The sample included 30 participants (6 males and 24 females), aged 20 to 54 years (mean 35.4 years, SD 9.2), education 10 to 19 years (mean 14.8 years, SD 2.1). Apart from primary diagnoses, a summary of the participants’ demographic and diagnostic information is presented in Table 1. Moreover, 15 participants continued to receive pharmacotherapy during the trial (4 fluvoxamine, 2 escitalopram, 1 sertraline, 1 paroxetine hydrochloride, 1 duloxetine, 1 mirtazapine, 1 trazodone, 1 ethyl loflazepate, 1 alprazolam, and 1 clotiazepam).

### Primary Outcomes

There were significant reductions for each symptoms of obsession-compulsion (Y-BOCS=−6.2; Cohen *d*=0.74; 95% CI −9.4 to −3.0; *P*=.002), panic (PDSS=−5.6; Cohen *d*=0.89; 95% CI −9.83 to −1.37; *P*=.02), and social anxiety (LSAS=−33.3; Cohen *d*=1.10; 95% CI −59.62 to −7.49; *P*=.02). Of the participants with OCD, 20% (2/10) showed a treatment response, whereas 40% (4/10) went into remission [75]. Of the participants with PD, 60% (6/10) showed a treatment response and 50% (5/10) went into remission [76]. Of the participants with SAD, 44% (4/9) showed a treatment response and 22% (2/9) went into remission [73].

### Secondary Outcomes

**Figure 2:**
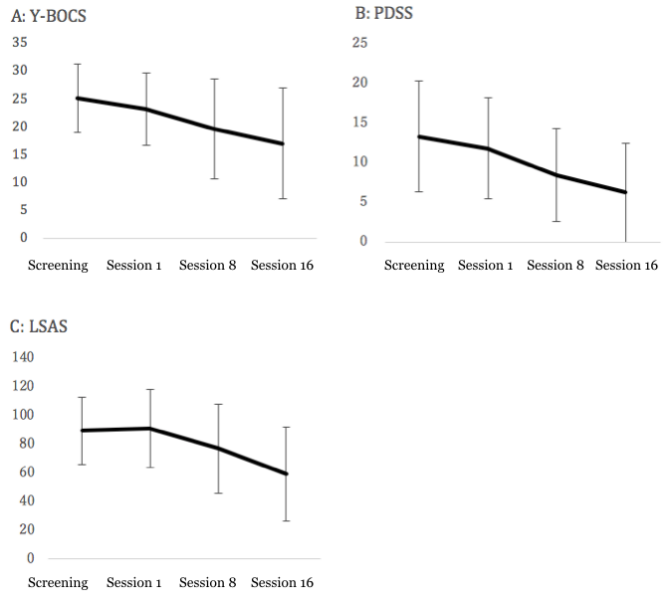
Change of primary outcomes. LSAS: Liebowitz Social Anxiety Scale; PDSS: Panic Disorder Severity Scale; Y-BCOS: Yale-Brown Obsessive-Compulsive Scale.

Figure 2 shows the change in the primary outcomes. Table 2 shows the mean change in the EQ-5D scores, at 16 sessions from baseline. The adjusted mean changes of the EQ-5D for all of the 3 disorders was 0.0336 (95% Cl −0.0198 to 0.0869; *P*=.21), which showed that it was not significant and showed a small effect size (Cohen *d=-* 0.202).

**Figure 3:**
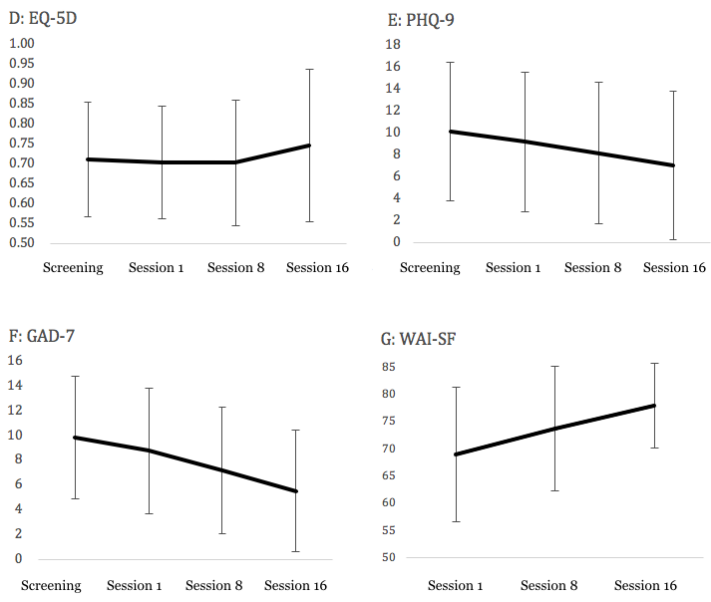
Change of secondary outcomes. EQ-5D-5L: EuroQol-5 Dimension; GAD-7: Generalized Anxiety Disorder-7; PHQ-9: Patient Health Questionnaire-9; WAI-SF: Working Alliance Inventory-Short Form.

Figure 3 also shows the change in the secondary outcomes. The mean changes in the PHQ-9 and GAD-7 scores reflected significant decreases in total participants for the 3 disorders (Table 3). The mean change in the WAI-SF reflected a significant increase for the total sample. Table 4 shows participants’ satisfaction with and preferences for videoconference-delivered CBT. As the ratings “very satisfied” and “satisfied” were combined, the majority of participants (86%, 25/29) reported being satisfied with videoconference-delivered CBT. In that case, the ratings “slightly prefer videoconference-delivered CBT,” “prefer videoconference-delivered CBT,” and “clearly prefer videoconference-delivered CBT” were combined; 83% (24/29) of the participants preferred videoconference-delivered CBT to face-to-face CBT. Conversely, 7% (2/29) of them preferred face-to-face CBT to videoconference-delivered CBT.

**Table 1:** Clinical and demographic characteristics of participants (N=30).

Characteristics		All	OCD^a^	PD^b^	SAD^c^
Age (years), mean (SD)		35.4 (9.2)	37.7 (6.9)	38.8 (9.8)	29.7 (8.6)
**Sex, n (%)**					
	Male	6 (20)	2 (6)	0 (0)	4 (13)
	Female	24 (80)	8 (27)	10 (33)	6 (20)
Employment, n (%)		11 (36)	5 (17)	2 (6)	4 (13)
Combined pharmacotherapy, n (%)		15 (50)	6 (20)	5 (17)	4 (13)
Videophone use experience, n (%)		15 (50)	4 (13)	6 (20)	5 (17)
**Comorbid disorders, n (%)**					
	Depression	5 (17)	1 (3)	1 (3)	3 (10)
	Panic/agoraphobia	2 (6)	2 (6)	N/A^d^	N/A
	PTSD^e^	1 (3)	N/A	1 (3)	N/A
	Alcohol dependence	1 (3)	N/A	N/A	1 (3)
	Bulimia nervosa	1 (3)	N/A	N/A	1 (3)
	GAD^f^	3 (10)	2 (6)	N/A	1 (3)

^a^OCD: obsessive-compulsive disorder.

^b^PD: panic disorder.

^c^SAD: social anxiety disorder.

^d^N/A: not applicable.

^e^PTSD: posttraumatic stress disorder.

^f^GAD: generalized anxiety disorder.

**Table 2:** Mean change in EuroQol-5 Dimension score.

Disorder	Mean change (95% CI)	*P* value (paired *t* test)	*P* value (*F* test)
All (n=29)	0.0336 (−0.0198 to 0.0869)	.21	N/A^a^
OCD^b^ (n=10)	0.0488 (−0.0577 to 0.1553)	.33	.91
PD^c^ (n=10)	0.0305 (−0.0393 to 0.1003)	.35	.91
SAD^d^ (n=9)	0.0201 (−0.1188 to 0.1591)	.75	.91

^a^N/A: not applicable.

^b^OCD: obsessive-compulsive disorder.

^c^PD: panic disorder.

^d^SAD: social anxiety disorder.

**Table 3:** Mean changes in secondary outcomes.

Measures and Disorder		Mean change, *t* value (95% CI)	*P* value (paired *t* test)	*P* value (*F* test)
**PHQ-9^a^**				
	All (n=29)	−1.72 (−3.26 to −0.19)	.03	N/A^b^
	OCD^c^ (n=10)	−1.70 (−4.52 to 1.12)	.21	.27
	PD^d^ (n=10)	−0.30 (−1.65 to 1.05)	.63	.27
	SAD^e^ (n=9)	−3.33 (−7.56 to 0.89)	.11	.27
**GAD-7^f^**				
	All (n=29)	−3.03 (−4.57 to −1.49)	<.001	N/A
	OCD (n=10)	−.50 (−6.37 to −0.63)	.002	.68
	PD (n=10)	−2.10 (−3.43 to 0.77)	.006	.68
	SAD (n=9)	−3.56 (−8.02 to 0.91)	.10	.68
**WAI-SF^g^**				
	All (n=29)	4.14 (1.24 to 7.04)	.007	N/A
	OCD (n=10)	3.00 (−1.77 to 7.77)	.19	.85
	PD (n=10)	4.80 (−0.42 to 10.02)	.07	.85
	SAD (n=9)	4.67 (−2.66 to 11.99)	.18	.85

^a^PHQ-9: Patient Health Questionnaire-9.

^b^N/A: not applicable.

^c^OCD: obsessive-compulsive disorder.

^d^PD: panic disorder.

^e^SAD: social anxiety disorder.

^f^GAD-7: Generalized Anxiety Disorder-7.

^g^WAI-SF: Working Alliance Inventory-Short Form.

**Table 4:** Satisfaction and preference.

Answer options		n (%)
**Satisfaction**		
	Very dissatisfied	0 (0)
	Dissatisfied	0 (0)
	Slightly dissatisfied	1 (3)
	Neutral	0 (0)
	Slightly satisfied	3 (10)
	Satisfied	9 (31)
	Very satisfied	16 (55)
**Preference**		
	Clearly prefer face-to-face	0 (0)
	Prefer face-to-face	1 (3)
	Slightly prefer face-to-face	1 (3)
	Neutral	3 (10)
	Slightly prefer videoconference-delivered CBT^a^	10 (34)
	Prefer videoconference-delivered CBT	6 (21)
	Clearly prefer videoconference-delivered CBT	8 (28)

^a^CBT: cognitive behavioral therapy.

### Adverse Events

A total of 3 patients reported adverse events, including depression relapse, headache, and feeling of exhaustion. The depressive symptoms of 1 SAD patient with depressive disorder worsened between the ninth and tenth sessions, when he was travelling with his friend. We identified this as a serious adverse event at the tenth session. He wanted to decline continuing with videoconference-delivered CBT and dropped out of the trial at that time. At 6 months after he received pharmacotherapy from his psychiatrist, he recovered the depressive episode. In addition, 1 PD participant reported a headache at the fourth session but recovered in the same day. Furthermore, 1 OCD patient reported a feeling of exhaustion after the 4th session but recovered in the same day.

## Discussion

### Principal Findings

This study examined the feasibility of videoconference-delivered CBT in adult patients with mild to severe OCD, PD, and SAD. Interventions based on CBT were conducted for each group divided by primary diagnosis, and examination of symptom improvement and acceptance of patients was conducted before and after the intervention. We use different criteria for each disease looking at the rate of responders to treatment (defined as a 35% reduction in Y-BOCS obsessive-compulsive symptoms, a 40% reduction in PDSS panic symptoms, and a 31% reduction in LSAS social anxiety symptoms); patients’ satisfaction were also confirmed by using therapeutic alliance and patients’ treatment acceptance. Improvement of the symptoms was confirmed in 3 disorders; it was found that the therapeutic alliance was achieved at a high level, and patients’ satisfaction was extremely high. Therefore, this study showed the feasibility of ICBT with real-time support of therapists to Japanese patients except for depression [86].

### Feasibility of Videoconference-Delivered Cognitive Behavioral Therapy

Regarding the other primary outcomes, the calculated Cohen *d* for pre- to posttreatment were 0.74 for the Y-BOCS, 0.89 for the PDSS, and 1.10 for the LSAS. The Cohen *d* scores were classified as medium and large. Though it is difficult to compare these studies because the characteristics of the patients and/or the methods were different, these results seem similar to those of our face-to-face CBT studies [59-61]. These medium and large effect sizes were also found in the previous studies of videoconference-delivered CBT (Cohen *d*=1.4-2.5) [50-52]. A previous videoconference-delivered CBT study for OCD reported that the treatment response rate was 60% (6/10). According to a systematic review about face-to-face CBT studies conducted between 2000 and 2014, the response rates were 43.3% for OCD, 53.2% for PD, and 45.3% for SAD [87]. The response rates in this study were 20% (2/10) for OCD, 60% (6/10) for PD, and 44% (4/9) for SAD. Previous videoconference-delivered CBT studies reported that the remission rate for PD was 81% (9/11) [52] and that for SAD was 54% (13/24) [50]. The remission rates in our studies were 40% (4/10) for OCD, 50% (5/10) for PD, and 22% (2/9) for SAD. Although comparisons of these results must be done with caution, the response and remission rates of this study were comparable with those in the previous studies of in-person CBT and videoconference-delivered CBT. In the future, it will be necessary to verify the effectiveness of our videoconference-delivered CBT through a randomized controlled trial or noninferiority trial in comparison with face-to-face CBT or videoconference-delivered CBT with different methodology.

There was no significant change (*P*=.21) in the EQ-5D scores for all of the 3 disorders, the calculated Cohen *d* from pre- to posttreatment was −0.202 and classified as small. In addition, there were no significant differences in changes of the EQ-5D score among the 3 disorder groups (*P*=.91). As described by the previous reports [88], our findings suggested that the EQ-5D was responsive in videoconference-delivered CBT for OCD, PD, and SAD.

For the PHQ-9, a significant reduction between pre- and posttreatment was observed for the entire sample (*P*=.03) There were no significant differences in the PHQ-9 changes among the 3 disorders (*P*=.27). The effect size for the 3 disorders was small (Cohen *d*=0.27). After dividing each disorder, the effect sizes ranged from small to medium (OCD: Cohen *d*=0.23; PD: Cohen *d*=0.07; SAD: Cohen *d*=0.64). As for the GAD-7, a significant reduction pre- and posttreatment was observed for the entire sample (*P*<.001). Although the change reflected a medium effect size for the entire sample (Cohen *d=* 0.61), all were medium for each disorder (OCD: Cohen *d*=0.75; PD: Cohen *d*=0.79; SAD: Cohen *d*=0.67). There were significant differences in changes between the OCD and PD groups (OCD: *P*=.002; PD: *P*=.006; SAD: *P*=.10) at week 16. A previous study reported a response rate in GAD-7 of 50.9% following a computerized CBT program, for 1062 adults who had GAD-7 scores of 10 or more at baseline [89] including 75 patients with GAD, 47 with PD, 40 with SAD, and 18 with PTSD. In this study, the treatment response rate for the GAD-7 was 45% (13/29). The response rates of PHQ-9 and GAD-7 in this study were similar to those in a prior study [89]. Taken together, our results suggest that videoconference-delivered CBT for OCD, PD, and SAD might secondarily ameliorate symptoms for generalized anxiety and depression.

More than half of the participants (55%, 16/29) who completed the videoconference-delivered CBT reported the highest level of satisfaction (“very satisfied”) with treatment via videoconferencing, whereas 31% (9/29) reported that they were “satisfied” (the second highest level). In other words, 86% (25/29) of participants reported being satisfied with videoconference-delivered CBT. These results are consistent with those of previous studies [50,90]. Furthermore, 83% (24/29) of the participants preferred videoconference-delivered CBT to face-to-face CBT. Conversely, 7% (2/29) preferred face to face CBT to videoconference-delivered CBT. Taken together, these results indicated that videoconference-delivered CBT was generally accepted by Japanese participants with OCD and anxiety disorders.

The therapeutic alliance indicated by the total scores of WAI-SF significantly improved throughout the treatment, from 68.9 (SD 12.3) at week 1 to 77.9 (SD 7.7) at week 16 (*P*=.007). The mean scores of the WAI-SF items were 5.7 (SD 0.97) at pretreatment, 6.1 (SD 0.95) at midtreatment, and 6.5 (SD 0.63) at posttreatment. These results were comparable with those of a previous study on videoconference-delivered CBT, where the WAI-SF scores increased from 5.22 (SD 0.42) to 5.60 (SD 0.90) in patients with SAD [50] and were 5.80 (SD 0.90) in patients with OCD [51]. Furthermore, these therapeutic alliance scores were comparable with those in a previous study on in-person CBT, where the WAI-SF scores increased from 5.78 (SD 0.94) to 5.93 (SD 0.90) in patients with PD and from 5.32 (SD 0.87) to 5.57 (SD 0.85) in patients with SAD [90]. Considering that lower alliance is known to be associated with dropout [90], the high alliance scores in this study can explain low dropout rate (3%, 1/30).

The dropout rate of this study was 3% (1/30), as 97% (29/30) completed the videoconference-delivered CBT treatment. Dividing the 3 disorders, the dropout rate of 10% (1/10) of participants with SAD was comparable with that of a previous study of videoconference-delivered CBT (17%, 4/24) [50] as well as a previous meta-analysis of 587 studies of in-person CBT between 1990 and 2010 (18%) [91]. The dropout rate of 0% for OCD was comparable with that of a previous study of videoconference-delivered CBT (0%, 0/10) [51] as well as the results of a meta-analysis of studies on in-person CBT published between 1993 and 2014 (15%) [92]. The dropout rate of 0% for PD was similarly comparable with a previous study on videoconference-delivered CBT (0%, 0/11) [52] as well as a meta-analysis of in-person CBT studies published between 1993 and 2002 (12.7%) [93].

### Limitations

This study has some limitations, including its small sample size, lack of a control group, unstandardized outcomes (satisfaction/preference of videoconference-delivered CBT), and long-term follow-up. Without a placebo control group and pharmacotherapy group, it remains unknown whether the observed improvements in symptom severity were merely the natural course, a result of the drug, or the effect of the intervention. Future studies should employ psychological placebo conditions and pharmacotherapy conditions. Thus, a 3-armed randomized controlled trial comparing pill placebo as the control group, videoconference-delivered CBT patients on antidepressants, and videoconference-delivered CBT patients who are drug-free should be designed and performed. We have been conducting a randomized controlled trial that includes the pharmacotherapy condition to provide greater insight into this CBT for PD since December 2016. In addition, we intend to conduct similar trials for OCD and SAD in the near future.

### Conclusions

This study demonstrated the feasibility of CBT with real-time support by the therapist to remotely treat adult patients with symptoms of obsessive-compulsion or anxiety, examining the reduction in symptoms before and after the intervention, and patient acceptance. As it was found that the mutual relationship between therapists and patients can be built on a high level by patients and that patients felt satisfaction about remote treatment with real-time therapist support via videoconference, we believe that videoconference-delivered CBT can be easily implemented on a larger scale in present Japan where the internet is easily accessible. Future research should aim at increasing the reach of intervention and determining whether the intervention is indeed more approachable to people who are young patients or those with a low socioeconomic status. Related to this matter, because a patient’s understanding level and information communication skills probably influence the effectiveness of remote treatment, future studies should be made on designs that consider the contents of support of the therapist beyond the absence or presence of guides.

## Abbreviations

AMED: Agency for Medical Research and Development
CBT: cognitive behavioral therapy
DSM: Diagnostic and Statistical Manual of Mental Disorders
EQ-5D: EuroQol-5 Dimension
GAD-7: Generalized Anxiety Disorder-7
ICBT: internet-delivered CBT
LSAS: Liebowitz Social Anxiety Scale
OCD: obsessive-compulsive disorder
PD: panic disorder
PDSS: Panic Disorder Severity Scale
PHQ-9: Patient Health Questionnaire-9
PTSD: posttraumatic stress disorder
SAD: social anxiety disorder
WAI-SF: Working Alliance Inventory-Short Form
Y-BOCS: Yale-Brown Obsessive-Compulsive Scale
